# Impact of adenosine in controlled aortic root reperfusion on clinical outcomes among patients undergoing valvular heart surgery

**DOI:** 10.1051/ject/2026006

**Published:** 2026-09-15

**Authors:** Farshad Jalili Shahandashti, Naser Kachoueian, Mostafa Fattahi, Davoud Samaie, Maryam Ghanbari Garekani, Atefeh Taherkhani, Seyedehmasoumeh Seyedebrahimi, Saied Heydari Nia, Farhad Gorjipour, Fazel Gorjipour

**Affiliations:** 1 Rajaie Cardiovascular, Medical and Research Center Tehran Iran; 2 Shahid Beheshti University of Medical Sciences Tehran Iran; 3 Department of Allied Medicine, Babol University of Medical Sciences Babol Iran; 4 SUNY Upstate Medical University Syracuse NY USA; 5 Iran University of Medical Sciences Tehran Iran

**Keywords:** Adenosine, Aortic root reperfusion, Cardiac operation, Cardiopulmonary bypass

## Abstract

*Background*: Adenosine is a vital medication in cardiac surgery, particularly in valvular heart procedures. While its use has been linked to improved postoperative cardiac function in some studies, there remains significant uncertainty regarding the adenosine usage in aortic reperfusion phase. This lack of consensus poses challenges for surgeons, perfusionists, and anesthesiologists alike. This study aims to explore the impact of adenosine on clinical outcomes in patients undergoing valvular heart surgery. *Method*: This prospective randomized controlled trial was conducted over a three-month period. Sixty patients undergoing valvular heart surgery were enrolled using a continuous sampling method and randomly allocated into two equal groups of 30 patients each. The intervention group received adenosine-enriched aortic root reperfusion immediately prior to aortic declamping, while the control group underwent standard warm blood aortic root reperfusion. Both groups were matched for demographic and clinical characteristics to ensure comparability. *Results*: Results indicated no significant differences in mean cardiopulmonary bypass (CPB) time, aortic cross-clamping duration, or mechanical ventilation between the intervention and control groups. However, the intervention group that received adenosine had a higher rate of antiarrhythmic agent usage in the operating room (*P* < 0.05). Inotropic agent usage was similar in both groups during surgery and in the ICU. Additionally, laboratory parameters on the first day of ICU admission were comparable between groups. *Conclusion*: Results in the control group showed more favorable outcomes in terms of anti-arrhythmic drug usage, electroshock application, and arrhythmia prevalence. This study showed advantages for the standard warm blood aortic root reperfusion technique in managing post-operative cardiac rhythm disturbances, in comparison with the trial group.

## Introduction

Cardioplegia is commonly used to minimize myocardial damage during cardiac surgery, but both reversible and irreversible injuries, such as myocardial edema and reduced cardiac function, still occur due to ischemia-reperfusion injury during cardiac operations [1]. These complications might lead to cardiac dysrhythmia, a decrease in ejection fraction, cardiac arrest, and increased recovery time for patients undergoing cardiac operations. Appropriate cardioplegia can be considered an important preventive measure for these complications; however, the optimal composition of cardioplegia fluid for myocardial protection has not yet been determined [2]. Injection of warm blood before aortic root declamping can wash and remove anaerobic metabolites and improve myocardial function [3]. Warm blood injection has had a significant impact on Troponin-I levels and significantly decreased the need for inotrope agents and intra-aortic balloon pumps [3]. Despite these advantages of using warm blood, Rergkliang, in his study, reported that using warm blood did not cause significant changes in operation-related parameters [4]. Although recent studies have suggested some additives, such as Insulin, Nicorandil, and Amiodarone, for modification of cardioplegia solution, there is no defined instruction for using warm blood within the CPB (Cardiopulmonary Bypass) among patients undergoing cardiac operations [1].

Adenosine, as an antiarrhythmic agent, has been used in managing cardiac arrhythmias and has recently been suggested for cardioplegia solution with warm blood injection [5]. Adenosine improves cardiac rhythm and helps return to normal rhythm. Some studies have reported that adenosine during ischemia can increase ventricular function and ejection fraction and reduce myocardial damage size [6]. Recent advances in LC-MS/MS-based metabolomics (liquid chromatography – mass spectrometry-based metabolomics) have enabled detailed profiling of biomolecules such as adenosine, which provides the necessary insights into their cardioprotective effects and potential as a therapeutic agent that could enhance myocardial protection and improve clinical outcomes associated with cardiac surgeries [7, 8]. It seems that using adenosine in warm blood infusion during CPB can decrease cardiac operation complications. Accordingly, the present study was performed to assess the impact of reperfusion of the aortic root with and without adenosine among patients undergoing cardiac valve operations. In this study, we hypothesized that adenosine supplementation in warm blood reperfusion before aortic declamping provides superior myocardial protection compared with warm blood alone, by reducing ischemia-reperfusion–related myocardial injury and improving functional recovery.

## Materials and methods

We conducted a prospective randomized controlled trial involving a total of 60 patients undergoing valvular heart surgery in 2020, who were consecutively sampled and randomly allocated into two equal groups of 30 patients. The study protocol was approved by ethical research committee of Shahid Rajaie Cardiovascular, Medical and Research Institute (IR.RHC.REC.1399.41), and all participants signed informed consents. Study participants were selected via a consecutive sampling method, according to inclusion criteria. Inclusion criteria were EF > 35%, patients aged 18–65 years old, and primary valvular replacement or repair. Exclusion criteria were ventricular hypertrophy, preoperative inotrope usage, cardiac pacemaker (ICD), pulmonary hypertension (PAP > 65 mmHg), recent myocardial infarction, perioperative need for *intra*-*aortic balloon pump**,*** and cardioprotective mechanical devices.

Randomization was performed with an accidental number table and a random block of four samples, and participants were randomly allocated to trial and control groups. Participants in both groups received mild hypothermia (minimum temperature 32 °C) [9] and their acid-base balance was controlled by the Alpha-stat method. The anesthesiology conditions and CPB settings were the same for all study patients. Del Nido cardioplegia was used for cardiac arrest, and half of the first dose was repeated 60 min after the initial. At the end of the ischemic phase and before aortic declamping, patients in the trial group received warm blood with adenosine (150 μg/kg) directly through the cardioplegia delivery line using a roller pump, at a rate of 200 to 250 mL per minute, within a time frame of 5 to 10 min, under a pressure of 80 to 100 mmHg until sinus rhythm was restored. This was administered through the cardioplegia line using a roller pump as part of the trial intervention. In the control group, patients received warm blood, similar to the trial group, without adenosine. For the prevention of blood clotting, Heparin was injected (300–400 units/kg) to reach the ACT higher than 480 s.

Demographic and baseline variables, including age, sex, weight, operation type, and cardiac disorder, were gathered by a researcher-made checklist via the medical documents and records. The pre- and post-operation cardiac ejection fraction of the patients was assessed by an echocardiologist who was blinded to the study groups by echocardiography. CPB-related data were gathered within the study period by the research team. Some operation-related parameters, including spontaneous sinus rhythm resumption time, antiarrhythmic and inotrope drugs usage, CPB time, frequency of cardiac shock and pacemaker usage after CPB, and cardiac muscle damage, and liver and kidney function markers were measured as study outcomes. Other clinical variables such as the need for blood transfusion and introp agents were recorded by the patient’s medical documents.

## Statistical analysis

The data were analyzed using IBM SPSS Statistics software (Version 22.0; IBM Corp., Armonk, NY, USA). Normality of study data was analyzed, and mean/standard deviation and frequency/percentages were used to describe quantitative and qualitative variables in normal distribution and median (IQR1–IQR3) for nonparametric variables. Independent sample t-test and chi-square statistical tests compared quantitative and qualitative variables between two groups for parametric and *Mann*–*Whitney U* test for non-parametric variables. All *P*-values less than 0.05 were assumed to be significant results.

## Results

Sixty patients in the trial and control groups were entered into the analysis. Demographic and baseline variables were similar between patients of both groups (Table 1).

**Table 1:** Comparison of demographic and baseline variables between included patients.

Groups Variables	Trial group (*n* = 30)	Control group (*n* = 30)	*P*-value
Age (Mean ± SD)	50.6 ± 13.17	52.36 ± 13.57	0.62
Sex			0.30
Male	17 (56.7%)	13 (43.3%)	
Female	13 (43.3%)	17 (56.7%)	
Underlying disease			0.42
Diabetes	1 (3.3%)	7 (43.3%)	
Hypertension	7 (23.3%)	4 (13.3%)	
Hyperlipidemia	2 (6.7%)	5 (16.7%)	
Operation type			–
MVR	17 (56.6%)	15 (50.0%)	
AVR	9 (30.0%)	12 (40.0%)	
MVR-TV repair	1 (3.33%)	–	
MV repaire	1 (3.33%)	–	
TVR	1 (3.33%)	–	
TVR-AVR	1 (3.33%)	–	
PVR	–	1 (3.33%)	
Resection AV	–	1 (3.33%)	
MVR-AVR	–	1 (3.33%)	

Mean of the CPB time was similar between patients of the trial and control groups (80.87 ± 20.07 vs. 74.90 ± 21.43 min; *P* = 0.27). There was no significant difference between aortic cross clamp time among patients of the trial and control groups (53.40 ± 19.07 vs. 50.63 ± 16.81 min; *P* = 0.55). There was no significant difference between the two groups in Mechanical ventilation time (10.0 ± 4.0 vs. 8.87 ± 3.48 h; *P* = 0.25). Ejection fraction of included patients had no significant differences at pre- and post-CPB and intensive care unit times (Table 2).

**Table 2:** Comparison of ejection fraction between included patients at different measurement points.

Ejection fraction	Trial group (*n* = 30)	Control group (*n* = 30)	*P*-value
Preoperation			0.72
35–45	11 (36.6%)	9 (30.0%)	
50	13 (30.0%)	8 (26.7%)	
55–60	10 (33.3%)	13 (43.3%)	
Post operation			0.42
35–45	10 (33.3%)	3 (10.0%)	
50	10 (33.3%)	12 (40.0%)	
55–60	10 (33.3%)	15 (50.0%)	
ICU			0.95
50–55	11 (36.7%)	11 (36.7%)	
40–45	7 (23.3%)	8 (26.7%)	
15–40	12 (4.0%)	11 (36.7%)	
CPB (min)	80.87 ± 20.07	74.90 ± 21.43	0.27	
Cross Clamp (min)	53.40 ± 19.07	50.63 ± 16.81	0.55	
Mechanical Ventilation Time (day)	10.0 ± 4.0	8.87 ± 3.48	0.25	

Study patients in the trial group received significantly higher antiarrhythmic agents in the operating room compared with patients in the control group (14, 43.3% vs. 5, 16.7%; *P* = 0.024). Antiarrhythmic usage rate was similar in patients of both groups in the ICU, and only one patient in the control group received antiarrhythmic agents (*P* = 0.31). Inotrope agents’ usage rate was similar between patients of trial and control groups in the operating room (20, 66.7% vs. 24, 80%; *P* = 0.243) and ICU (10, 33.3% vs. 6, 20%; *P* = 0.25). Although cardiac shock usage was significantly higher among patients of the trial group in comparison with the control group, pacemaker usage rate was similar between patients of both groups (*P* = 0.99) (Table 3).

**Table 3:** Comparison of cardiac shock and pacemaker usage among patients of trial and control groups.

Variable		Trial group (*n* = 30)	Control group (*n* = 30)	*P*-value
Cardiac shock				0.001
Yes		13 (43.3%)	2 (6.7%)	
No		17 (56.7%)	28 (93.3%)	
Number of shock usage				0.004
0		17 (56.7%)	28 (93.3%)	
1		8 (26.7%)	1 (3.3%)	
2–5		5 (16.7%)	1 (3.3%)	
Pacemaker				0.99
Yes		4 (13.4%)	3 (10.0%)	
No		26 (86.7%)	27 (90.0%)	

The time of warm blood infusion among patients of both groups was similar. The type of cardiac resumption rhythm was significantly different among patients in the trial and control groups. According to that, Ventricular Tachycardia (VT), Ventricular Fibrillation (VF), and Dysrhythmia to sinus rhythm frequency were significantly higher among patients in the trial group (Table 4).

**Table 4:** Comparison of warm blood infusion time and cardiac rhythm return among patients of trial and control groups.

Variable	Trial group (*n* = 30)	Control group (*n* = 30)	*P*-value
Warm blood infusion time (min)			1.00
0–5	28 (93.3%)	28 (93.3%)	
5–10	2 (6.7%)	2 (6.7%)	
Cardiac rhythm resumption arrhythmia			0.012
Sinus Tachycardia	11 (36.7%)	16 (53.3%)	
VT	8 (26.7%)	4 (13.3%)	
VF	5 (16.7%)	0	
Dysrhythmia to sinus	4 (13.3%)	1 (3.3%)	
Junctional	1 (3.3%)	6 (20%)	
Sinus	1 (3.3%)	4 (13.3%)	
Returning time to cardiac rhythm (min)	2.94 ± 1.93	3.19 ± 1.72	0.59

In the first day to ICU entrance, laboratory parameters were similar between two groups (Table 5).

**Table 5:** Comparing laboratory parameters on the first day of ICU entrance among patients of trial and control groups.

Variable	Trial	Control	*P*-value
Troponin (ng/mL)	3.6 ± 0.93	3.1 ± 1.93	0.17
Ck-mb (ng/mL)	44.03 ± 16.64	39.81 ± 14.93	0.305
CPK (ng/mL)	320.83 ± 160.87	273.83 ± 180.20	0.174
LDH (U/L)	600.80 ± 229.12	577.27 ± 132.77	0.629
SGOT(U/L)	38.23 ± 17.41	36.07 ± 10.57	0.563
SGPT(U/L)	21.87 ± 15.80	18.50 ± 7.47	0.298
Cr (mg/dL)	0.0 (0.9–1.4)	1 (0.7–1.2)	0.06
BUN (mg/dL)	15.1 ± 5.44	16 ± 9.07	0.65

Ck-mb: Creatine Kinase-MB, CPK: Creatine Phosphokinase, LDH: Lactate Dehydrogenase, SGOT: Serum Glutamic-Oxaloacetic Transaminase Test, SGPT: Serum Glutamic-Pyruvic Transaminase, Cr: Creatinine, BUN: Blood Urea Nitrogen.

## Discussion

In the present study, 60 patients in both trial and control groups were included, and baseline variables were similar between study participants in the two groups. Duration of operation, aorta cross-clamping, ICU stay, and mechanical ventilation time were similar between the two groups. Our findings were similar to those reported by Givtaj et al. [10], which supports the validity of our results. In contrast to our results, Kalita et al. [11] reported that patients who used the traditional method without warm blood injection in the aortic root, in their mitral valve operation had significantly higher ICU stay time and therefore had higher operation-related complications. Although EF of included patients was decreased after the operation, there were no significant differences between the two groups. Although Givtaj et al. [10] had a similar result with our study, Ammar et al, in their study reported that patients who received adenosine before aortic declamping had better systolic and diastolic function [12].

We found that the time of cardiac rhythm resumption was similar between patients in the two groups. Elgariah et al., in their study, reported that in patients who received adenosine, the cardiac rhythm returning time and incidence of cardiac rhythm abnormalities were significantly lower than those of the control group [3]. The frequency of ventricular fibrillation and tachycardia was significantly higher among our patients in the trial group compared to those in the control group. In contrast to our findings, Sallam et al. found that patients who used adenosine had experienced a lower frequency of cardiac rhythm abnormalities and atrial and ventricular arrhythmia [13]. Ascione et al., reported that adenosine did not significantly influence the type of cardiac rhythm resumption [14]. Rergkliang et al., in their study, reported that patients who did not receive warm blood during the operation, need a lower time to cardiac rhythm resumption [4]. Our findings suggest that adenosine administration might impair cellular metabolic conditions and contribute to non-sinus cardiac rhythm and delayed rhythm recovery.

Antiarrhythmic agents’ usage in patients of the trial group was significantly higher than that of patients in the control group. On the other hand, the incidence of dysrhythmia in the operating room among patients of the trial group was higher than that of the control group. There were no significant differences in the incidence of arrhythmia among patients of both groups in the ICU. In contrast to our findings, Ammar et al., in their studies, reported that use of adenosine can decrease the incidence of postoperative arrhythmia [12]. Givtaj et al. reported that there was no considerable relationship between the incidence of arrhythmia and adenosine usage among patients undergoing cardiac operations [10]. Patients of both groups had similar inotropic usage rates in the operating room and ICU. Ascione et al. had similar findings to our study [14]. Khairy, P et al., reported that pediatric patients who did not receive adenosine had a significantly higher need for supportive and inotropic agents in comparison with patients who received adenosine during the operation [15].

Means of the Troponin and CK-MB, as two main myocardial damage markers, were similar between the patients of the two groups. On the other hand, the use of adenosine did not reduce postoperative troponin among patients. Ayman S et al. in their study reported that mean of troponin among patients who did not receive hot shot during their operation was significantly higher than that of other patients [13]. Elgariah et al. reported that the level of troponin among patients of the adenosine group was significantly lower than that of patients of the control group [3]. Cardiac enzymes are known as effective factors of cardiac function and can predict postoperative situations of patients undergoing cardiac operations. Although we had not seen any significant difference in postoperative troponin level between the two groups.

In our study, the frequency of cardiac electroshock applications among patients in the trial group was significantly higher than that in the control group. This finding can be related to the decline in cardiac function and increase in cardiac enzymes among patients in the trial group who received adenosine during their operation. Although in our study the mean level of cardiac enzymes did not show significant differences between the two groups, higher cardiac rhythm abnormalities and more antiarrhythmic drug usage in the operating room indicate that patients who received adenosine required more therapeutic interventions to improve their cardiac rhythm; and we hypothesize potential mechanisms including adenosine receptor desensitization, inter individual variability in response, and metabolic effects during reperfusion. Ammar et al. reported opposite results, noting that cardiac electroshock usage among patients who received adenosine was lower than in other patients [12]. Ascione et al. reported that the usage of cardiac electroshock was similar between patients of both groups [14]. It seems that theoretically, use of adenosine should reduce cardiac rhythm abnormalities after aortic decamping, and we need more studies in the cellular and clinical settings to confirm the impact of adenosine on the postoperative cardiac rhythm of patients. Use of adenosine in our study had no significant impact on the postoperative cardiac ejection fraction of patients. Givtaj et al.’s study had similar findings [10] and in contrast to our findings, Ammar et al., in their study, reported that patients who received adenosine had better postoperative systolic and diastolic functions [12].

## Limitation

Due to patient recruitment restrictions and ethical limitations, the current study included 30 participants per group. We agree that this sample size may have limited statistical power for detecting differences in clinical outcomes, and we now acknowledge the lack of power analysis as a limitation.

## Conclusion

In this randomized clinical study, adenosine administration during the reperfusion phase did not demonstrate a clear clinical advantage compared to the control group. Demographic variables, ejection fraction, ICU stay, and sinus rhythm recovery showed no significant differences between groups, while a higher rate of postoperative dysrhythmias was observed in the adenosine group. These findings suggest that adenosine, despite its well-documented cardio-protective mechanisms through A1 and A3 receptor activation, may not uniformly confer antiarrhythmic benefits and could potentially exert pro-arrhythmic effects under certain physiological conditions, such as receptor desensitization or individual dose-response variability. The present results emphasize the complexity of adenosine’s actions in myocardial reperfusion and highlight that timing, dosage, and patient-specific factors may critically determine its effects.

In conclusion, the findings of this study are specifically observed in patients undergoing heart valve surgery. These results highlight the clinical outcomes and implications of adenosine administration during the controlled aortic root reperfusion phase in this particular patient population

Given the relatively small sample size, the study may have been underpowered to detect subtle clinical benefits or adverse effects. Thus, larger randomized trials are warranted to confirm these observations and to clarify the risk-benefit balance of adenosine use during cardiac surgery, particularly in the reperfusion phase.

## Data Availability

The data supporting the findings of this study are available from the corresponding author upon reasonable request.
